# Comparing efficiency of health systems across industrialized countries: a panel analysis

**DOI:** 10.1186/s12913-015-1084-9

**Published:** 2015-09-25

**Authors:** Bianca K. Frogner, H.E. Frech, Stephen T. Parente

**Affiliations:** Department of Family Medicine, School of Medicine, University of Washington, 4311 11th Ave. NE, Suite 210, Box 354982, Seattle, WA 98195 USA; Department of Economics, University of California, Santa Barbara, 2127 North Hall, Mail Stop 9210, Santa Barbara, CA 93106 USA; Finance Department, Carlson School of Management, University of Minnesota, 321 19th St. South, 3-122, Minneapolis, MN 55455 USA

**Keywords:** International comparison, Health systems, Stochastic frontier analysis, Efficiency, Life expectancy

## Abstract

**Background:**

Rankings from the World Health Organization (WHO) place the US health care system as one of the least efficient among Organization for Economic Cooperation and Development (OECD) countries. Researchers have questioned this, noting simplistic or inappropriate methodologies, poor measurement choice, and poor control variables. Our objective is to re-visit this question by using newer modeling techniques and a large panel of OECD data.

**Methods:**

We primarily use the OECD Health Data for 25 OECD countries. We compare results from stochastic frontier analysis (SFA) and fixed effects models. We estimate total life expectancy as well as life expectancy at age 60. We explore a combination of control variables reflecting health care resources, health behaviors, and economic and environmental factors.

**Results:**

The US never ranks higher than fifth out of all 36 models, but is also never the very last ranked country though it was close in several models. The SFA estimation approach produces the most consistent lead country, but the remaining countries did not maintain a steady rank.

**Discussion:**

Our study sheds light on the fragility of health system rankings by using a large panel and applying the latest efficiency modeling techniques. The rankings are not robust to different statistical approaches, nor to variable inclusion decisions.

**Conclusions:**

Future international comparisons should employ a range of methodologies to generate a more nuanced portrait of health care system efficiency.

## Background

As key provisions of the 2010 Patient Protection and Affordable Care Act (ACA) roll out in 2014, researchers and policymakers will be asking whether the US health care system is gaining efficiency. The ACA will increase insurance coverage, which may increase workload of an already overburdened primary care workforce [1–3]. Simultaneous adoption of health information technology (or the use of computers to store, retrieve, share and use health care information, data and knowledge) spurred by the 2009 Health Information Technology for Economics and Clinical Health (HITECH) Act has been reported to add to workforce burdens, although the long-run goal is to reduce administrative waste, and duplicative and unnecessary services [4–6]. As these major changes take effect, efficiency may be an elusive goal in the near-term for the US health care system. But even more elusive may be finding a methodology to monitor the efficiency of the US health care system.

The World Health Organization (WHO) published one of the most recognized rankings of health care system performance in the 2000 report titled *Health Systems: Improving Performance* [7]. The US placed 37^th^ out of 191, behind Costa Rica, on overall health system performance. The report has been criticized for its objectives, confounding of social influences with health care system performance, poor data quality, and narrow scope in methodology [8–10]. Some of these critics re-estimated efficiency and rankings, using different approaches, which generally led to different rankings [9, 11, 12]. The US tended to rank higher in these newer studies, although none placed the US at the top.

Since the 2000 WHO report, several studies have focused on the efficiency of the health care systems of industrialized countries [13–18]. These studies vary in their choice of output measures and statistical approaches. The most common, although not universal conclusion, is that US still does not fare well compared to other member countries of the Organization for Economic Cooperation and Development (OECD).

Researchers continue to question whether the methodologies employed in all these aforementioned studies on efficiency and rankings are strong enough to make valid conclusions, and to what extent the limitations impact the conclusions [19, 20]. In this paper, we re-visit the question of whether the US health care system is efficient relative to other industrialized health care systems. Our research goals are to apply the recommendations from past studies and capitalize on advancements in modeling techniques to produce new results. We discuss the sensitivity of our results to variations of the model specifications, and how these variations affect the ranking of the US.

In the next section we discuss the dataset used for our analysis and our approach to choosing the variables in our model. We then present the statistical approaches used in the past, and the more recently available approaches, to rank countries. We compare the results of the various permutations of input and output variables along with statistical approaches, and discuss the implications of our results in the Discussion section.

## Methods

### Data

We primarily use data from the 2013 OECD Health Data, which is a comprehensive health systems dataset on 34 members of the OECD [21]. Although some data are available as far back as 1960, for our variables of interest, the most complete set of data runs from 1990 through 2010 (21 years). All data used in this study are available for public use. This study is not human subject research, and is not subject to Institutional Review Board review. The set of countries included in our analyses varied due to the choice of variables and estimation approaches. Our final set of countries includes 25 out of the 34 countries, and excludes Canada, Chile, France, Greece, Ireland, Netherlands, Portugal, Switzerland, and Turkey, due to missing data.

Some of our country time series contains gaps of varying length. All international comparisons face this major limitation given that not all countries report all variables for all years; thus we have an unbalanced panel. We did not impute values for missing data. Instead, we dropped observations with missing data, which we considered in our sensitivity analyses.

Our output measure is life expectancy in order to match the approach used in other health system ranking studies [14, 15, 18]. We estimated three output measures: 1) total life expectancy, 2) life expectancy at age 60 for males, and 3) life expectancy at age 60 for females. Total life expectancy by gender was not available, nor was a combined gender measure of life expectancy at age sixty. A limitation of total life expectancy is that causes of death in the earlier years are more strongly related to social differences than to health care (e.g. drug overdose, accidental injuries, perinatal conditions, and homicide) [22]. Given this limitation, we also included life expectancy at age sixty (for females and males) as an output measure since the health care system plays an increasingly important role at later stages in life and this care may have an impact on the remaining life expectancy.

We tested numerous combinations of 33 input variables based on theory and empirical evidence estimating each of our three output measures – total life expectancy, life expectancy at age 60 for males, and life expectancy at age 60 for females. Generally, we tested metrics for health care resources, service utilization, lifestyle and risk behaviors, environmental factors, and economic factors. Although the OECD Health Data includes a wide range of potential input variables, our final choice of variables was in part determined due to availability of data and frequency of data reporting (Table 1). Our final models included a combination of the following 11 input variables: physicians per 1,000 capita (for which we manually inserted two recent data points for the US from OECD cited data sources), magnetic resonance imaging (MRI) and computed tomography (CT) scanners per million population, years of potential life lost (YPLL) from self-harm, homicide, transportation, and alcohol use per 100,000 capita, calories consumed per capita per day (adjusted for average population height in centimeters), percent of the population fifteen years and older who smoked daily, cubic tons of total nitrogen dioxide (adjusted for square meter of land), and gross domestic product (GDP) per capita (in purchasing power parities and deflated to 2005 dollars) [23–25]. We applied the adjustments to calories for height as well as to nitrogen dioxide to land mass in effort to provide more meaningful comparisons across countries. We tested several other input variables in our sensitivity analyses, which we discuss in our Results section.

**Table 1:** Percent data missing input variables for study countries

Categories	Variables	% Data Missing	
Health care		All	US
		*N* = 525	*N* = 21
Resources:	Total hospital beds per 1,000 capita	19.1	4.8
Capital	Acute care beds per 1,000 capita	17.3	4.8
Labor	Total health employees per 1,000 capita	23.2	0.0
	Total active doctors per 1,000 capita	16.2	0.0
	Total general practitioners (GPs) per 1,000 capita	46.7	14.3
	Total specialist doctors per 1,000 capita	54.5	14.3
	Total professional active nurses per 1,000 capita	64.0	42.9
Utilization	Total hospital discharges per 1,000 capita	34.7	4.8
	Average length of stay in acute care (days)	15.4	0.0
	Total doctor visits per capita	29.5	38.1
Service intensity	Ratio of GPs to specialists	55.2	14.3
	Ratio of nurses to doctors	70.1	42.9
Technology	MRI scanners per million population	49.1	52.4
	CT scanners per million population	47.8	66.7
Risk Factors			
Chronic disease^a^	Percent mortality due to chronic disease	0.0	0.0
	Percent years potential life lost (YPLL) due to chronic disease	0.0	0.0
Lifestyle and behaviors	Calories consumed per capita		
	Per day	36.8	14.3
	Per day per centimeter	41.7	14.3
	Liters of alcohol consumed by individuals 15 years or older		
	Per day	26.5	4.8
	Per day per centimeter	34.3	4.8
	Average number of cigarettes smoked per smoker per day	62.7	4.8
	Percent of population (15 years and older) smoking daily	46.7	4.8
	Percent of youth (15 to 24 years old) smoking daily	64.6	4.8
	Grams of tobacco consumed per capita	39.2	0.0
	Percent population self-reported as obese	60.8	28.6
	YPLL from assaults per 100,000 capita	7.1	9.5
	YPLL from self-harm per 100,000 capita	6.7	9.5
	YPLL from transportation per 100,000 capita	6.7	9.5
	YPLL from alcohol use per 100,000 capita	9.9	9.5
	YPLL from drug use per 100,000 capita	49.7	52.4
Environmental	Total nitrogen dioxide (measured in cubic tons and deflated to 1990 values)		
	Per capita	27.8	0.0
	Per square meter of land	5.9	0.0
	Mobile (% total)	7.6	0.0
	Stationary (% total)	7.6	0.0

^a^Chronic disease includes mental health diseases, cancer, diabetes, chronic obstructive pulmonary disease, kidney disease, cerebrovascular disease, acute myocardial infarction, and ischemic heart disease.

### Estimation approach

We estimated technical efficiency of a health care system by its ability to maximize the life expectancy of its population using a minimum amount of health care and non-health care inputs. We compared three estimation approaches: 1) country fixed effects (FE) model, 2) country and time FE model, and 3) stochastic frontier analysis (SFA). We applied each estimation approach to predict each of the three output measures of life expectancy (i.e., total, at age 60 for males, and at age 60 for females) as a function of various permutations of the input variables described above. In this paper, we present the results from our final 36 models.

FE models are common approaches for estimating technical efficiency of health care systems. To identify efficient countries, we compared the size of the country FE component of the residual. The most efficient country is defined as the country with the largest positive residuals. In other words, efficient countries have higher-than-predicted life expectancies given the same set of inputs compared to other countries. The least efficient country has the most negative residuals, or lower-than-predicted life expectancies. A series of F-tests suggested that FE was more appropriate than an ordinary least squares (OLS) approach. Breusch-Pagan Lagrange Multiplier tests suggested that random effects was also more appropriate than an OLS approach. Hausman test results support the use of FE over RE although the variance matrix was not positive definite for these models because the Hausman test requires strict conditions for homoscedasticity, which our model most likely do not have. To address heteroscedasticity, we used robust standard errors.

SFA has been used for efficiency comparisons in health care [26]. SFA has appeared only once in the peer-reviewed literature to compare efficiency of health care in OECD countries; however, the comparison focused on hospitals [27]. SFA is a parametric approach that imposes a functional form (i.e., Cobb-Douglas production function estimated as a log-linear model) on the relationship between the inputs and outputs [28]. We tried to estimate a SFA models with maximum likelihood techniques [29], but after trying multiple versions of the model with different arrays of variables, we could not reach convergence. Instead we used a less data intensive least squares approach, which has been shown to provide reliable results [30, 31].

The SFA is often preferred over a non-parametric approach, data envelopment analysis (DEA), which is limited in the number of allowable inputs and does not separate out “noise” from the inefficiency term [32]. SFA assumes a random disturbance term that is normally distributed and a technical inefficiency term that has a strictly non-negative distribution. We assumed a half-normal distribution of the inefficiency term, which is a common yet narrow assumption. The choice in the distribution of the inefficiency term has been found to have a negligible influence on empirical results [12]. We allowed technical inefficiency to vary over time rather than stay constant as in the FE models. In SFA, the most efficient country has an inefficiency residual term that is equal to zero; all other countries with an inefficiency residual term less than zero are considered less efficient.

For ranking, within a model, the country with the largest positive residual (or zero in the SFA model) is ranked number one. The remaining countries are ordered based on the distance of their residual from the number one ranked country; the larger the difference in residuals from the number one country, the lower the country rank.

## Results

In Table 2, we show the US rankings for 12 out of 36 models - four different sets of input variables by three different estimation approaches – estimating total years of life expectancy at birth of the population. Japan ranks first for over half of the models presented in Table 2, and for 75 % of the models estimating female life expectancy at age sixty. The US rank varies considerably going from as high as sixth out of 25 to as low as thirteenth out of 14 countries. Across all 36 models, we find a generally dispersed picture with the US ranking near the center (Tables 3, 4 and 5).  The US never ranks higher than fifth out of all 36 models, but is also never the very last ranked country though it was close in several models. The SFA estimation approach produces the most consistent lead country, but the remaining countries did not maintain a steady rank.

**Table 2:** Country ranks when estimating total life expectancy (years)

Estimation approach		Model A	Model B	Model C	Model D
Fixed Effect w/ Country	US Rank	6/25	16/20	9/14	13/14
	Top Rank Country	New Zealand	Slovenia	Japan	Japan
	Sample Size	394	203	133	133
Fixed Effects w/ Country and Year	US Rank	12/25	14/20	7/14	8/14
	Top Rank Country	Japan	Israel	Australia	Australia
	Sample Size	394	203	133	133
Stochastic Frontier Analysis	US Rank	15/23	10/18	11/13	12/13
	Top Rank Country	Japan	Japan	Japan	Japan
	Sample Size	393	201	133	133
Independent Variables	Total physicians per 1,000 capita	x	x	x	x
	YPLL from assaults per 100,000 capita	x	x	x	x
	YPLL from self-harm per 100,000 capita	x	x	x	x
	YPLL from transportation per 100,000 capita	x	x	x	x
	YPLL from alcohol use per 100,000 capita	x	x	x	x
	MRI scanners per million population		x		
	CT scanners per million population		x		
	Calories consumed per capita per day (adjusted for average population height in cm)			x	x
	Percent of population (15 years and older) smoking daily			x	x
	Total (mobile + stationary) nitrogen dioxide (measured in cubic tons) per square mile of land			x	x
	GDP per capita, PPP (Deflated to 2005 Dollars)				x

NOTE: Smoking variable is lagged by 5 years; *YPLL* = Years of potential life lost; *CT* = computed tomography; *MRI* = magnetic resonance imaging; GDP = Gross Domestic Product; PPP = Purchasing Power Parities

**Table 3:** Country ranks when estimating total life expectancy (years)

Country	Model A			Model B			Model C			Model D		
	FE	FE full	SFA	FE	FE full	SFA	FE	FE full	SFA	FE	FE full	SFA
Australia	5	4	4	5	3	3	7	1	3	4	1	4
Austria	17	13	10	15	12	4	13	8	7	12	7	10
Belgium	12	9	11				5	12	10	11	12	9
Czech Republic	22	21	19	17	14							
Denmark	21	19	16	14	13	13	12	13	12	14	14	13
Estonia	24	25	23	18	18	17						
Finland	8	15	13	10	10	7	11	6	6	7	6	6
Germany	19	14	9				6	11	8	10	11	8
Hungary	25	24	22	19	19	18						
Iceland	11	5	2	11	5	2	8	2	2	5	2	2
Israel	18	8	7	6	1	5						
Italy	9	2		13	4							
Japan	3	1	1	8	2	1	1	4	1	1	4	1
Korea	2	17	18	4	11	12	2	10	13	2	9	11
Luxembourg	4	10	12	7	8	8						
Mexico	16	20	21	9	15	16						
New Zealand	1	6	8	2	6	6	3	3	5	3	3	5
Norway	15	11	6									
Poland	20	22	20	12	16	15	14	14		8	13	
Slovak Republic	23	23		20	20							
Slovenia	7	18	17	1	9	11						
Spain	10	3	5									
Sweden	14	7	3				10	5	4	6	5	3
United Kingdom	13	16	14	3	7	9	4	9	9	9	10	7
United States	6	12	15	16	14	10	9	7	11	13	8	12

Note: *FE* = country fixed effects; *FE Full* = country and time fixed effects; *SFA* = stochastic frontier analysis

**Table 4:** Country ranks when estimating male life expectancy at age 60 (years)

Country	Model A			Model B			Model C			Model D		
	FE	FE full	SFA	FE	FE full	SFA	FE	FE full	SFA	FE	FE full	SFA
Australia	5	3	5	5	5	4	13	1	2	9	1	3
Austria	17	16	8	16	13	7	10	9	7	14	9	8
Belgium	11	13	13				4	13	11	8	13	11
Czech Republic	22	22	20	17	17	15						
Denmark	21	19	17	15	15	9	7	12	12	12	12	12
Estonia	23	25	23	19	19	17						
Finland	9	15	16	11	11	10	14	7	10	13	6	10
Germany	19	17	10				5	10	8	6	10	6
Hungary	24	24	22	18	18	18						
Iceland	10	4	1	10	6	1	11	2	1	7	2	1
Israel	18	6	3	6	1	3						
Italy	15	9		14	8							
Japan	3	1	4	8	3	2	2	3	4	1	4	4
Korea	2	18	19	7	12	14	1	11	13	2	11	13
Luxembourg	7	11	15	9	10	11						
Mexico	4	5	11	1	2	12						
New Zealand	1	2	9	2	3	5	8	4	5	3	3	5
Norway	16	12	7									
Poland	20	21	21	12	16	16	9	14		5	14	
Slovak Republic	25	23		29	29							
Slovenia	8	20	18	4	14	13						
Spain	13	7	6									
Sweden	14	10	2				12	5	3	10	5	2
United Kingdom	12	14	14	3	7	6	3	8	9	4	8	7
United States	6	8	12	13	9	8	6	6	6	11	7	9

Note: *FE* = country fixed effects; *FE Full* = country and time fixed effects; *SFA* = stochastic frontier analysis

**Table 5:** Country ranks when estimating female life expectancy at age 60 (years)

Country	Model A			Model B			Model C			Model D		
	FE	FE full	SFA	FE	FE full	SFA	FE	FE full	SFA	FE	FE full	SFA
Australia	5	3	2	7	3	2	7	1	4	4	1	6
Austria	13	11	9	14	10	6	10	8	5	9	7	7
Belgium	11	5	6				3	11	2	11	12	2
Czech Republic	23	22	21	18	18	16						
Denmark	22	20	18	17	15	13	14	14	13	14	14	13
Estonia	20	23	22	16	17	15						
Finland	8	13	8	10	8	3	9	5	3	6	5	3
Germany	17	14	11				6	10	9	10	11	8
Hungary	24	25	23	19	19	18						
Iceland	14	8	7	13	9	4	12	4	8	7	4	10
Israel	21	15	13	12	4	11						
Italy	10	3		11	2							
Japan	1	1	1	2	1	1	1	2	1	1	2	1
Korea	2	16	17	5	13	8	2	9	10	3	9	5
Luxembourg	4	7	10	4	5	5						
Mexico	12	19	19	6	14	17						
New Zealand	3	6	12	3	6	10	4	3	6	2	3	4
Norway	15	10	5									
Poland	16	21	20	9	16	14	8	12		5	8	
Slovak Republic	25	24		20	20							
Slovenia	9	17	16	1	11	9						
Spain	6	2	2									
Sweden	18	12	4				13	6	7	8	6	9
United Kingdom	19	18	15	8	7	12	11	13	11	13	13	11
United States	7	9	14	15	12	7	5	7	12	12	10	12

Note: *FE* = country fixed effects; *FE Full* = country and time fixed effects; *SFA* = stochastic frontier analysis

In Tables 6 and 7, we see that physicians per capita is consistently significant in the country only FE models, but loses significance in the country and time FE and SFA models. YPLL from transportation is the only variable to be significant across the three estimation approaches in Model A, but it loses significance with the addition of technology, environmental, lifestyle, and economic variables. Generally no consistent pattern emerges in the significance of the input variables, and the significance of the inputs appear to be sensitive to the variation in the set of countries in the analysis.

**Table 6:** Coefficients estimating total life expectancy (years) – models A and B

	Model A			Model B		
	FE	FE full	SFA	FE	FE full	SFA
Total physicians per 1,000 capita	0.065***	0.010	0.001	0.049**	0.018	0.004
	(0.017)	(0.013)	(0.003)	(0.016)	(0.013)	(0.005)
YPLL from assaults per 100,000 capita	−0.003	−0.003	−0.000	0.003	−0.001	−0.000
	(0.005)	(0.002)	(0.000)	(0.003)	(0.001)	(0.000)
YPLL from self-harm per 100,000 capita	−0.012	0.004	−0.001	−0.005	0.008	−0.000
	(0.009)	(0.010)	(0.002)	(0.006)	(0.005)	(0.002)
YPLL from transportation per 100,000 capita	−0.039***	−0.018*	−0.005***	−0.018*	−0.010	−0.001
	(0.008)	(0.008)	(0.001)	(0.008)	(0.006)	(0.001)
YPLL from alcohol use per 100,000 capita	−0.001	0.000	−0.004***	−0.005	−0.004*	−0.003***
	(0.003)	(0.002)	(0.000)	(0.004)	(0.002)	(0.001)
MRI scanners per million population				0.010***	0.003	−0.002*
				(0.002)	(0.002)	(0.001)
CT scanners per million population				0.011***	0.006**	−0.001
				(0.003)	(0.002)	(0.001)
Year						
1991		0.003			−0.002	
		(0.002)			(0.003)	
1992		0.005*			−0.004	
		(0.002)			(0.003)	
1993		0.006			−0.005	
		(0.003)			(0.004)	
1994		0.009**			−0.004	
		(0.003)			(0.004)	
1995		0.009*			−0.002	
		(0.003)			(0.005)	
1996		0.013***			0.002	
		(0.003)			(0.005)	
1997		0.017***			0.004	
		(0.004)			(0.006)	
1998		0.018***			0.006	
		(0.005)			(0.006)	
1999		0.020***			0.010	
		(0.004)			(0.006)	
2000		0.026***			0.014*	
		(0.005)			(0.007)	
2001		0.029***			0.018*	
		(0.005)			(0.007)	
2002		0.031***			0.019*	
		(0.005)			(0.007)	
2003		0.032***			0.019*	
		(0.005)			(0.007)	
2004		0.036***			0.023**	
		(0.006)			(0.008)	
2005		0.038***			0.025**	
		(0.006)			(0.008)	
2006		0.041***			0.027**	
		(0.006)			(0.008)	
2007		0.043***			0.027**	
		(0.006)			(0.009)	
2008		0.043***			0.028**	
		(0.007)			(0.01)	
2009		0.046***			0.031**	
		(0.007)			(0.01)	
2010		0.048***			0.035**	
		(0.009)			(0.01)	
Constant	4.587***	4.397***		4.389***	4.321***	
	(0.072)	(0.063)		(0.071)	(0.045)	
Observations	394	394	393	203	203	201
Adjusted R-squared	0.820	0.910		0.874	0.935	

Note: *FE* = country fixed effects; *FE Full* = country and time fixed effects; *SFA* = stochastic frontier analysis; YPLL = Years of potential life lost; CT = computed tomography; MRI = magnetic resonance imaging

*p < 0.05, **p < 0.01, ***p < 0.001

**Table 7:** Coefficients estimating total life expectancy (years) – models C and D

	Model C			Model D		
	FE	FE full	SFA	FE	FE full	SFA
Total physicians per 1,000 capita	0.061*	0.041*	0.007	0.047	0.039*	0.008
	(0.028)	(0.018)	(0.007)	(0.022)	(0.017)	(0.006)
YPLL from assaults per 100,000 capita	0.000	0.000	−0.000	0.001	0.000	−0.000
	(0.001)	(0.001)	(0.001)	(0.001)	(0.001)	(0.001)
YPLL from self-harm per 100,000 capita	−0.002	0.001	−0.002	−0.002	0.001	0.000
	(0.004)	(0.004)	(0.001)	(0.005)	(0.005)	(0.002)
YPLL from transportation per 100,000 capita	−0.026*	−0.013	−0.002	−0.021*	−0.013	−0.002
	(0.009)	(0.007)	(0.001)	(0.007)	(0.008)	(0.001)
YPLL from alcohol use per 100,000 capita	−0.003*	−0.001	0.000	−0.001	−0.000	0.000
	(0.001)	(0.002)	(0.001)	(0.002)	(0.002)	(0.001)
Calories consumed per capita per day (adjusted for average population height in cm)	0.048	0.002	−0.012	0.065	0.009	−0.022
	(0.025)	(0.034)	(0.012)	(0.038)	(0.034)	(0.012)
Percent of population (15 years and older) smoking daily	−0.025*	−0.004	0.002	−0.017*	−0.004	0.002
	(0.011)	(0.006)	(0.002)	(0.007)	(0.006)	(0.002)
Total (mobile + stationary) nitrogen dioxide (measured in cubic tons) per square mile of land	−0.008	0.018*	0.004	0.004	0.018*	0.003
	(0.009)	(0.006)	(0.003)	(0.007)	(0.006)	(0.003)
GDP per capita, PPP, deflated to 2005 dollars				0.038***	0.008	0.017**
				(0.007)	(0.007)	(0.006)
Year						
1991		0.000			0.000	
1992		0.000			0.000	
1993		0.000			0.000	
1994		0.000			0.000	
1995		−0.029***			−0.027***	
		(0.004)			(0.005)	
1996		−0.026***			−0.024***	
		(0.004)			(0.004)	
1997		−0.023***			−0.022***	
		(0.004)			(0.004)	
1998		−0.021***			−0.019***	
		(0.003)			(0.003)	
1999		−0.017***			−0.016***	
		(0.003)			(0.003)	
2000		−0.015			−0.014***	
		(0.002)			(0.002)	
2001		−0.012			−0.011***	
		(0.001)			(0.002)	
2002		−0.011***			−0.010***	
		(0.001)			(0.001)	
2003		−0.010***			−0.009***	
		(0.001)			(0.011)	
2004		−0.006***			−0.006***	
		(0.001)			(0.001)	
2005		−0.004***			−0.004***	
		(0.001)			(0.001)	
2006		−0.002			−0.002	
		(0.001)			(0.001)	
2007		0.000			0.000	
2008		0.000			0.000	
2009		0.000			0.000	
2010		0.000			0.000	
Constant	4.579***	4.292***		3.952***	4.178***	
	(0.219)	(0.153)		(0.193)	(0.173)	
Observations	133	133	133	133	133	133
Adjusted R-squared	0.890	0.946		0.915	0.946	

Note: *FE* = country fixed effects; *FE Full* = country and time fixed effects; *SFA* = stochastic frontier analysis; Smoking variable is lagged by 5 years; YPLL = Years of potential life lost; GDP = Gross Domestic Product; PPP = Purchasing Power Parities

*p < 0.05, **p < 0.01, ***p < 0.001

We note that the inclusion of GDP per capita (Model D) does not add much explanatory power to the models and loses its significance with the addition of time fixed effects. Including GDP raises concerns about endogeneity. A long literature exists pursuing this line of causation that generally states that when health status improves, workers may be more productive, raising GDP [33]. To gauge how rankings perform relative to a measure of real resources in health care, we plotted the ranks against our main health resources variable - number of physicians per 1,000 capita. In Figure 1, we plot the rankings from the SFA estimation of Model A and find a generally dispersed picture with the US near the center. The pattern generally holds across all models. 

### Sensitivity analysis

We tried alternative input measures to estimate life expectancy. We found that our results were not sensitive to the height and land mass adjustments to calories and pollution, respectively. Although physicians per 1,000 capita is a very common metric in estimating health system efficiency, we lost about twenty percent of our sample by using this metric. We tested total health employment as an alternative, but the ranking results for the countries in both models remained about the same. We chose physicians over total health employment given the more definitive nature of counting physicians versus the more ambiguous definition of a “health employee”; for example, some countries include social assistance workers as health employees. We tested number of physician visits but the US was missing one-third of the years of observations. We tried physician-to-nurse and general practitioner-to-specialist ratios, but given that countries did not provide consistent years of data for these variable sets, many countries were excluded. We tested the inclusion of acute care beds as well as total number of hospital discharges (two variables that were also highly correlated with each other), but found that the measure was not significant in any of our models and its inclusion did not noticeably change the rankings. We tested alcohol consumption as an alternative to YPLL from alcohol consumption, but again generally found the same results. We ideally would have more measures for chronic disease and related risk factors, but data availability was limited. For example, obesity would seem to be an important measure, but each country collects the data differently (i.e., measured versus self-report) and the data has only been reported for a few years. We also would like to lag cigarette use over more years, but again the years of data availability is limited. Despite these data limitations, our findings remained consistent. The rankings varied based on the set of countries, years and input variables included.

We also tried different estimation approaches. In a residual analysis, we saw a clear time trend in the random disturbance from the FE models. We tested and found evidence for serial correlation in our FE models. Although the traditional corrections for serial correlation are available, the latest literature advises against these corrections in the absence of strong prior on the nature of the serial correlation and the use of robust standard errors instead [34]. With the SFA model, we see a more randomly dispersed pattern to the error term although there is a slight upward drift. While we control for heteroskedasticity in the FE models, we do not have a mechanism to correct for heteroskedasticity in the SFA models, which may bias our coefficients but not necessarily the relative rankings. SFA also requires at least two time observations for each country, so we would lose Italy and the Slovak Republic.

## Discussion

Our study sheds light on the fragility of health system rankings by using a large panel and applying the latest efficiency modeling techniques. While we find that the US tends to be in the middle to lower third of country rankings, the US is never the last ranked country and is occasionally near the top ranked countries. Rankings vary considerably when different variables are included in the model and also when different statistical approaches are used. Among the statistical approaches presented in this study, the SFA model is the preferred given the distribution properties of the error term and the allowance of technical efficiency to vary over time. As a result, the rankings resulting from SFA may be considered superior over the other statistical approaches.

Although health care spending measures are often included in discussions around health system performance, we exclude this variable from our analysis since it conflates the discussion around prices versus quantity. We included measure of quantity (e.g., health service utilization and resources), but the OECD Health Data does not have any measures for prices (e.g., medical price index or medical care purchasing power parities exchange rate). Given the complexities of what price captures such as differences in labor markets, health care financing and organization and quality of care, health care spending seriously overstates the real resources used in health care in some countries, especially in the US [35].

There are three limitations with our analysis. First we were limited in the statistical methods that are possible, given data problems. Second, though we have better controls for environmental, lifestyle and behavioral risk factors than past literature, these controls are imperfect. Many important variables are not generally available or are not well measured. Third, while closer to measuring health care system efficiency than previous efforts, our results still capture efficiency differences that are beyond the health care system.

### Suggestions for an international data repository

The OECD has been a trusted source of data to compare industrialized countries. They have accomplished the significant and highly valuable task of harmonizing secondary data collected in different manners across countries with different capacities to share data. We put forth two recommendations for improvement. First, we recommend a permanent public repository of the data. Every year the OECD releases a new set of historical data, which includes revisions to older OECD releases. While this a tremendous asset, there is also an archival concern. Data or specific countries and years that may have been available in the past via the OECD data set are sometimes no longer to be found in new releases. These revisions need to be carefully tracked and documented to protect confidence in the reliability of the data.

Second, we recommend that the OECD invest in a federated data locater system where the location of independent data sources from countries are clearly identified for further analysis. The current process of manual harmonization of data sometime leaves out existing data. For example, the 2013 OECD Health Data did not include the number of physicians per 1,000 capita for the years of 2009 and 2010 for the US. Our team manually extracted these data points from the original source cited in the OECD in order to include these data points in our analysis.. Future analysts of OECD will be well served by data systems able locate and harmonize data automatically while allowing the data to be maintained and housed autonomously by member countries.

## Conclusion

In this study, we addressed criticisms that we and others have made regarding the validity of international rankings by using a wide array of statistical methods and more inclusive data. Our results do not refute prevailing belief in the literature that the US is a big health care spender and does not consistently deliver the highest quality health care or achieve the best health outcomes. Depending on the estimation approach and choice of variables, however, the US does rank high in technical efficiency. The lack of consistency in our results suggest that users of the existing set of published rankings proceed with caution.

With the change in US investment in the health economy following the ACA and HITECH Act, change in efficiency will be critical to measure. Doing so consistently must be a priority for accurate comparison of countries health care systems in the future.
